# Preclinical Evaluation of the GRPR-Targeting Antagonist RM26 Conjugated to the Albumin-Binding Domain for GRPR-Targeting Therapy of Cancer

**DOI:** 10.3390/pharmaceutics12100977

**Published:** 2020-10-16

**Authors:** Ayman Abouzayed, Hanna Tano, Ábel Nagy, Sara S. Rinne, Fadya Wadeea, Sharmishtaa Kumar, Kristina Westerlund, Vladimir Tolmachev, Amelie Eriksson Karlström, Anna Orlova

**Affiliations:** 1 Department of Medicinal Chemistry, Uppsala University, 751 83 Uppsala, Sweden; Ayman.Abouzayed@ilk.uu.se (A.A.); sara.rinne@ilk.uu.se (S.S.R.); Fadya.Wadeea.6129@student.uu.se (F.W.); 2 Department of Protein Science, School of Engineering Sciences in Chemistry, Biotechnology and Health, KTH Royal Institute of Technology, AlbaNova University Center, 106 91 Stockholm, Sweden; htano@kth.se (H.T.); abelna@kth.se (Á.N.); shakum@kth.se (S.K.); krw@kth.se (K.W.); ameliek@kth.se (A.E.K.); 3 Department of Immunology, Genetics and Pathology, Uppsala University, 751 85 Uppsala, Sweden; vladimir.tolmachev@igp.uu.se; 4 Research Centrum for Oncotheranostics, Research School of Chemistry and Applied Biomedical Sciences, Tomsk Polytechnic University, Tomsk 634050, Russia; 5 Science for Life Laboratory, Uppsala University, 751 05 Uppsala, Sweden

**Keywords:** prostate cancer, gastrin-releasing peptide receptor, RM26, albumin-binding domain, targeted therapy, gastrin-releasing peptide receptors (GRPR) antagonist

## Abstract

The targeting of gastrin-releasing peptide receptors (GRPR) was recently proposed for targeted therapy, e.g., radiotherapy. Multiple and frequent injections of peptide-based therapeutic agents would be required due to rapid blood clearance. By conjugation of the GRPR antagonist RM26 (D-Phe-Gln-Trp-Ala-Val-Gly-His-Sta-Leu-NH_2_) to an ABD (albumin-binding domain), we aimed to extend the blood circulation of peptides. The synthesized conjugate DOTA-ABD-RM26 was labelled with indium-111 and evaluated in vitro and in vivo. The labelled conjugate was stable in PBS and retained specificity and its antagonistic function against GRPR. The half-maximal inhibitory concentration (IC_50_) of ^nat^In-DOTA-ABD-RM26 in the presence of human serum albumin was 49 ± 5 nM. [^111^In]In-DOTA-ABD-RM26 had a significantly longer residence time in blood and in tumors (without a significant decrease of up to 144 h pi) than the parental RM26 peptide. We conclude that the ABD-RM26 conjugate can be used for GRPR-targeted therapy and delivery of cytotoxic drugs. However, the undesirable elevated activity uptake in kidneys abolishes its use for radionuclide therapy. This proof-of-principle study justified further optimization of the molecular design of the ABD-RM26 conjugate.

## 1. Introduction

Prostate cancer is one of the most commonly diagnosed and deadliest cancers in men worldwide [1]. A lot of research has been focused on identifying novel prostate cancer cell targets, raising the sensitivity towards diagnosing prostate cancer, including detection of distant metastases, and expanding the therapeutic options for patients.

The gastrin-releasing peptide receptor (GRPR), a G-protein coupled receptor, is a member of the bombesin receptor family. GRPR is normally expressed in different organs such as the pancreas and the stomach [2], and is overexpressed in various cancers, including prostate and breast cancers. GRPR overexpression in prostate cancer is androgen dependent and is found in 63–100% of primary prostate cancer samples and in more than 50% of lymph node and bone metastases, but not in hyperplastic and benign prostate cells [3,4]. Overexpression of GRPR is high in the early stages of prostate cancer (but decreases with disease progression when tumors dedifferentiate into higher grade) and in androgen-insensitive and spread metastatic lesions [5,6,7].

These findings established GRPR as an important target for diagnostic imaging of prostate cancer and, possibly, for therapeutic use. A limited clinical study in patients with prostate cancer (*n* = 11) reported high sensitivity, specificity, and accuracy (88%, 81% and 83%, respectively) in the detection of primary lesions, and a sensitivity of 70% for the detection of metastatic lymph nodes [8]. Numerous bombesin analogues have been developed to target GRPR [9], but antagonists have recently been favored over agonists for receptor targeting [10]. Activation by agonists causes receptor downregulation [11] and is accompanied by physiological actions that are not limited to cell proliferation [12].

RM26 (D-Phe-Gln-Trp-Ala-Val-Gly-His-Sta-Leu-NH_2_) is an extensively studied GRPR antagonist [13]. It binds to GRPR with high affinity and shows favorable pharmacokinetics when linked to different macrocyclic chelators using PEG linkers [14,15,16]. In clinics, [^68^Ga]Ga-NOTA-PEG_3_-RM26 has recently been evaluated for GRPR imaging in patients with prostate and breast cancers. [^68^Ga]Ga-NOTA-PEG_3_-RM26 could efficiently detect primary tumors and distant metastases, and was well tolerated by patients [17,18,19].

Other GRPR antagonists have demonstrated inhibition of cell proliferation in vitro, and multiple injections of therapeutic doses of the GRPR antagonist RC-3095 in a phase I trial were well tolerated; however, no follow-up studies were reported [20]. Antagonists to GRPR labelled with lutetium-177 were evaluated in preclinical studies on mice bearing prostate cancer xenografts, demonstrating a promising therapeutic efficacy with extended survival of the mice receiving the radiolabelled peptide [21,22]. The GRPR antagonist [^177^Lu]Lu-RM2 was studied for the treatment of patients with metastatic castration-resistant prostate cancer by delivering at least one therapy cycle of the GRPR-targeting radioligand [23]. The therapy was deemed safe with no reported adverse effects and with the pancreas being the dose-limiting organ. However, due to rapid blood clearance of the labelled peptide, multiple frequent injections are required to deliver an appropriate dose to the tumor.

Extending the biological half-life of the GRPR-targeting ligand is a way to minimize the number of injections and to prolong the bioavailability of the ligand for binding to tumor cells. One approach of extending the biological half-life is by conjugating an albumin-binding domain (ABD) that would extend the biological half-life of the targeting agent. The engineered albumin-binding domain ABD035, derived from streptococcal protein G, has a femtomolar affinity for human serum albumin (HSA) [24]. ABD035 and related variants have been conjugated to several proteins for increases in vivo half-life, and the conjugates were considered safe and non-immunogenic, and have also been used in clinical studies with no reported adverse effects [25].

In this study, we aimed to develop and perform preclinical evaluation of the GRPR antagonist RM26 conjugated to ABD035 and assess the possibility for using the ligand in GRPR-targeting therapy for cancer treatment. The chelator DOTA (1,4,7,10-tetraazacyclododecane-1,4,7,10-tetraacetic acid) was coupled to the *N*-terminus of ABD for radiolabeling with indium-111 for in vitro and in vivo characterization. DOTA is a convenient chelator for stable coordination of indium-111, an isotope with relatively long half-life of 2.6 d that allows for the examination of biodistribution over several days.

## 2. Materials and Methods

Prostate carcinoma cell line PC-3 (GRPR positive) was purchased from ATCC, Manassas, VA, USA. The cells were maintained in RPMI-1640 media supplemented with 10% fetal bovine serum (FBS), 1% penicillin–streptomycin (PEST) (100 IU/mL penicillin, 100 µg/mL streptomycin), and 1% 2 mM L-glutamine (L-Glut), all purchased from Biochrom AG, Berlin, Germany, and incubated at 37 °C in 5% carbon dioxide gas and 95% air.

The activity content was measured using a 2840 automated gamma counter Wizard^2TM^ (PerkinElmer, Waltham, MA, USA). Instant thin-layer chromatography (ITLC) results were analyzed using Cyclone^®^ Plus (PerkinElmer). Statistical analyses were performed by unpaired, two-tailed *t*-tests using GraphPad Prism 8 for Windows (GraphPad Software, San Diego, CA, USA); *p* values below 0.05 were considered significant.

### 2.1. General Peptide Synthesis

Peptides were produced by solid phase peptide synthesis (SPPS) with a fully automated microwave-assisted synthesis instrument (Biotage^®^-Initiator Alstra, Uppsala, Sweden). The amino acid monomers were Fmoc-protected, and as solid support, a Rink amide ChemMatrix resin (Biotage^®^) was used. Amino acids were purchased from Advanced Chemtech (Louisville, KY, USA), Novabiochem (St. Louis, MO, USA) and Sigma Aldrich (St. Louis, MO, USA). For a scale of 0.1 mmol peptide, 200 mg of resin was used. The resin loading was 0.5 mmol/g, and a five-times molar excess of amino acid was used in each cycle. For the coupling reaction, equimolar concentrations of DIC (*N,N*-diisopropylcarbodiimide, Sigma Aldrich, St. Louis, MO, USA)/Oxyma (ethyl (2*Z*)-2-cyano-2-hydroxyiminoacetate, Merck, Kenilworth, NJ, USA) (1:1) were used to activate the amino acids. Double coupling steps were applied for selected amino acid residues. 

The temporary protecting group Fmoc was cleaved with 20% piperidine (Sigma Aldrich) in NMP (1-methyl-2-pyrrolidone, Merck). The non-reacted amino groups were capped with acetic anhydride (Honeywell Fluka, Charlotte, NC, USA)/DIEA (*N,N*-diisopropylethylamine, Sigma Aldrich) after each round of coupling. The amino acid side chains were protected with the following protecting groups: tBu (*tert*-butyl) for Ser and Tyr; Pbf (2,2,4,6,7-pentamethyldihydrobenzofuran-5-sulfonyl) for Arg; Trt (trityl) for Asn and His; Boc (*tert*-butyloxycarbonyl for Lys; and OtBu (*tert*-butyl ester) for Glu and Asp. The orthogonal protecting group Mtt (4-methyltrityl) was used for selected Lys residues.

#### 2.1.1. Synthesis of DOTA-ABD-Cl 

The 46 amino acid ABD was synthesized by SPPS as described in the previous paragraph. Double coupling steps were applied for selected amino acid residues: Asn9, Lys14, Tyr15, Tyr21, Arg23 and Asn26. A DOTA chelator (DOTA-tris(tBu)ester, CheMatech) was manually coupled to the N-terminus using DIC/Oxyma (1:1)-activated coupling chemistry. After the conjugation of DOTA, the Mtt-protected Lys14 was manually deprotected by 10 × 2 min treatment of the peptide–resin with a reaction solution of DCM/TFA/TIS (dichloromethane, VWR (Radnor, PA, USA)/trifluoroacetic acid, Alfa Aesar, Kandel, Germany)/triisopropylsilane, Sigma Aldrich) at a 94:1:5 ratio. The deprotected Lys residue was acylated with chloroacetic acid (Sigma Aldrich) (10 equivalents) in the presence of *N,N*-dicyclohexylcarbodiimide (DCC, Aldrich) (5 equivalents) as an activator and DIEA (10 equivalents) as a base for 1 h at room temperature. All manual coupling and deprotection steps were monitored by ninhydrin tests and repeated if necessary.

The final product was cleaved from the resin, and the side chains were deprotected with a mixture of TFA/TIS/H_2_O 95:2.5:2.5 for 3 h. The crude peptide was extracted with H_2_O/*tert*-butyl-methyl ether (Merck) with a 1:1 ratio, and the water phase containing the peptide was lyophilized. The correct molecular weight was confirmed after the synthesis by MALDI-TOF mass spectrometry (MALDI TOF/TOF analyzer, Sciex, (Applied Biosystems, Foster City, CA, USA).

#### 2.1.2. Synthesis of RM26

The bombesin analogue RM26 was synthesized by SPPS as described above. Fmoc-statine was incorporated without side chain protection. After the synthesis of the first nine amino acids, the PEG_4_ linker (Fmoc-15-amino-4,7,10,13-tetraoxapentadecacanoic acid, ChemScene, Chemtronica AB, Sollentuna, Sweden) and the N-terminal thiol group-containing mercaptopropionic acid (S-trityl-β-mercaptopropionic acid, Peptides International, Gardner, MA, USA) were coupled manually to the RM26 peptide (D-Phe-Gln-Trp-Ala-Val-Gly-His-Sta-Leu-NH_2_) in sequential steps. The coupling and deprotection steps were monitored by ninhydrin tests and repeated if necessary. As a last step of the synthesis, a final piperidine deprotection was performed in order to reverse possible acylation of the statine side chain. After the final cleavage from the resin using a mixture of TFA/TIS/H_2_O/EDT 94:1:2.5:2.5 and an ether extraction step, the correct product was analyzed and verified with MALDI-TOF.

#### 2.1.3. Purification of DOTA-ABD-Cl and RM26

DOTA-ABD-Cl and RM26 were purified by reversed-phase HPLC (RP-HPLC) (Agilent 1200 series, Agilent Technologies, Santa Clara, CA, USA) on a semi-preparative column (5 μm, 9.4 × 250 mm Zorbax 300SB-C_18_, Agilent Technologies). A gradient of 20–60% acetonitrile in H_2_O with 0.1% TFA was used as the mobile phase. The total running time was 30 min with a flow rate of 3 mL/min, and a 40 °C column temperature was applied in order to maximize the degree of separation. The peaks were collected and analyzed by MALDI-TOF to identify the correct product. The fractions containing the correct product were pooled and lyophilized.

#### 2.1.4. Conjugation of DOTA-ABD-Cl to RM26

Purified DOTA-ABD-Cl and RM26 were conjugated through formation of an alkyl thioester between the chloroacetyl and thiol functional groups on the two molecules. The reaction was performed as described by Lindgren, et al. [26] in a ligation buffer containing 10 mM EDTA (ethylenediaminetetraacetic acid) in phosphate buffered saline (PBS, 60%, pH 8) with acetonitrile as co-solvent (40%). The pH of the reaction was set to 8–8.5 with NaOH (5%). A two times molar excess of RM26 was used. The total peptide concentration was approximately 2 mg/mL. To avoid the formation of disulfide-linked RM26 dimers and to reduce dimers already present in the solution, TCEP (tris(2-carboxyethyl)phosphine) (10 mM TCEP for 0.2–1 mg/mL protein) was added to RM26 prior to the conjugation reaction.

The final product was purified by reversed-phase high-performance liquid chromatography (RP-HPLC) with a gradient of 20–60% acetonitrile in H_2_O with 0.1% TFA as the mobile phase over 30 min with a flow rate of 3 mL/min and 40 °C column temperature. The peaks were collected and analyzed by MALDI-TOF mass spectrometry, and the correct molecular weight was confirmed with electrospray ionization-mass spectrometry (ESI-MS) (Thermo Ultimate3000, Thermo Fisher Scientific, Waltham, MA, USA, Bruker Impact II, Bruker Daltonics, Billerica, MA, USA). The purity of the conjugate was analyzed with an analytical RP-HPLC column (3.5 μm, 4.6 × 150 mm Zorbax 300SB-C_18_, Agilent Technologies) using the same conditions as described for the purification of the product. The protein concentration of the samples used for surface plasmon resonance (SPR) analysis and circular dichroism (CD) spectroscopy was determined by quantitative amino acid analysis (Alphalyse, Odense, Denmark).

#### 2.1.5. Expression and Purification of Recombinant ABD Control Protein

Methods for expression and purification of recombinant ABD can be found in detail in the Supplementary Materials Information. In brief, ABD035 was expressed as a thioredoxin fusion protein using the pET32a expression vector in BL21 (DE3) star *Escherichia coli* cells. After overnight autoinduction, the cells were lysed by sonication, and the His_6_-tagged fusion protein was purified using immobilized-metal ion chromatography (IMAC) purification. Following enterokinase-His_6_ (Sino Biological, Beijing, China) cleavage, the now untagged ABD035 protein was purified by collecting the unretarded flow-through from a second IMAC round. The purity and molecular weight of the purified ABD035 were confirmed using MALDI-TOF and SDS-PAGE.

### 2.2. Circular Dichroism

The secondary structures of ABD035, DOTA-ABD-Cl and DOTA-ABD-RM26 were determined by circular dichroism spectroscopy (Chirascan, Applied Photophysics, Leatherhead, UK). All CD spectra were obtained at 20 °C using a protein concentration of 0.2 mg/mL in 20 mM potassium phosphate buffer with 100 mM KCl pH 7.4. To estimate the percentage of helicity in each construct, the measured ellipticity (θ_obs_) was first converted to the mean residue ellipticity (MRE) using the following formula MRE = θ_obs_/10 × l × C × *n*, where l is the pathlength in cm, C is the peptide concentration in the molar, and *n* is the number of residues in each construct. From the mean residue ellipticity at 222 nm, MRE_222_, the fraction helix, F_H_, was calculated using the following formula, F_H_ = (MRE_222_ − [θ]_C_)/([θ]_H_ − [θ]_C_). [θ]_H_ and [θ]_C_ are given by [θ]_H_ = 40,000 × (1 − (2.5/*n*) + 100 × T, and [θ]_C_ = 640 − 45 × T. T is given in °C, here 20, and *n* is the number of residues in each construct (Scholtz 1991).

### 2.3. Surface Plasmon Resonance (SPR) Experiments

The interaction between DOTA-ABD-RM26 and HSA was investigated using Biacore T200 (GE Healthcare Life Sciences, Uppsala, Sweden). HSA was immobilized to 660 RU at a dextran surface on a Series S Sensor Chip CM5 chip (GE Healthcare Life Sciences, Uppsala, Sweden). Immobilization was performed using standard (1-ethyl-3-(3-dimethylamino) propyl carbodiimide, hydrochloride/*N*-hydroxysuccinimide (EDC/NHS) amine coupling procedures. After immobilization of the ligand, remaining unreacted NHS esters were deactivated by injection of 1 M ethanolamine. One surface was activated followed by deactivation and used as a reference, and another surface was immobilized with L1CAM-Fc (Sino Biological) as a control in all SPR experiments. All runs were performed with phosphate buffered saline with 0.5% Tween®-20 (PBST), pH 7.4 as running buffer. DOTA-ABD-RM26 at seven concentrations (0.27, 0.82, 2.47, 7.4, 22.2, 66.7 and 200 nM) was injected onto HSA for 150 sec at a flow rate of 30 µL/min. Dissociation was allowed for 7200 s (2 h) followed by surface regeneration by injection of 10 mM HCl. As comparison, unconjugated ABD035 and DOTA-ABD-Cl were injected to the surface at the same concentrations, using the same flow rate, association time and dissociation time. Kinetic parameters were calculated using a 1:1 Langmuir binding model in the Biacore T200 Evaluation software. All runs were performed in duplicates.

### 2.4. Radiolabeling and In Vitro Stability of DOTA-ABD-RM26

The DOTA-ABD-RM26 conjugate was radiolabeled with indium-111 by adding [^111^In]InCl_3_ (2.3–19.7 MBq) to 14–21 µg of DOTA-ABD-RM26 (2–3 nmol) and 80 µL of ammonium acetate buffer (0.2 M, pH 5.5). The reaction mixture was incubated at 85 °C for 30 min, and the radiochemical yield was determined by radio ITLC using 0.2 M citric acid buffer for elution. The radiolabeled conjugate was purified using NAP-5 size-exclusion columns. The labelling stability was tested by adding 1000-fold molar excess of EDTA or PBS to [^111^In]In-DOTA-ABD-RM26, and the percentage of indium-111 release was determined using radio ITLC at 1, 4 and 24 h at room temperature. For some of the experiments, HSA was bound to the purified [^111^In]In-DOTA-ABD-RM26 by adding 10-fold molar excess of HSA and allowing the conjugation to proceed for 1 h at room temperature.

Parental peptide DOTA-RM26 was labelled with indium-111 according to previously described procedure [14] with quantitative yield (determined by HPLC) and used in experiments without purification.

### 2.5. In Vitro Specificity Assay

The in vitro specificity assay was performed on PC-3 cells (GRPR positive). One day before the experiment, the cells (5 × 10^5^ cells/well) were plated on 6-well plates. At the time of the assays, the cells were washed, and 500 nM/well of DOTA-ABD-RM26 in the presence of HSA or 250 nM/well of DOTA-RM26 were added to the blocked wells in triplicates. The blocking was allowed to proceed for 10 min at room temperature; then, 20 nM/well of [^111^In]In-DOTA-ABD-RM26 plus HSA was added to non-blocked wells and to the wells blocked with either DOTA-ABD-RM26 plus HSA or DOTA-RM26. The dishes were incubated for 1 h at 37 °C followed by washing the cells and treatment with trypsin–EDTA to the cells detached. The cells were then collected, and the radioactivity content was measured using a gamma counter.

### 2.6. Competitive Binding (IC_50_) Assay

PC-3 cells (5 × 10^5^ cells/well) were plated on 12-well plates one day before the competitive binding assays. Three conjugates (^nat^In-DOTA- RM26, ^nat^In-DOTA-ABD-RM26 and ^nat^In-DOTA-ABD-RM26 with addition of HSA) were used to compete with [^111^In]In-NOTA-PEG_4_-RM26 for GRPR. DOTA-RM26 and DOTA-ABD-RM26 were loaded with stable indium by adding 3-fold molar excess of InCl_3_ to each conjugate, and the reactions proceeded as described earlier. On the day of the experiment, the cells were washed, and 1 nM of [^111^In]In-NOTA-PEG_4_-RM26 was added to each well along with a series of concentrations ranging between 0−10 µM of the competing conjugate. The dishes were incubated at 4 °C for 5 h. The cells were then washed and treated with trypsin–EDTA until cell detachment. The cells were collected, and the radioactivity content was measured using a gamma counter.

### 2.7. Cellular Processing Assay

For the cellular processing assay, PC-3 cells (5 × 10^5^ cells/well) were plated on 35 × 10 mm dishes two days before the experiment. On the day of the experiment, the cells were washed, and 20 nM/well of [^111^In]In-DOTA-ABD-RM26 plus HSA were added. At predetermined time points of 1, 2, 4, 8 and 24 h, the cells were washed, and the dishes were placed on ice. The membrane-bound fraction was separated by adding 1 mL of 0.2 M glycine buffer containing 0.15 M NaCl and 4 M urea (pH 2) and incubation on ice for 5 min. The solution was collected, and the cells were washed with more glycine buffer that was collected as well. To collect the cells and determine the internalized fraction, 0.5 mL of 1 M NaOH was added followed by incubation at 37 °C for 30 min. The cells were scraped, and the solution was collected after washing with additional 1 M NaOH. The radioactivity content was then measured on a gamma counter.

### 2.8. In Vivo Targeting Specificity and Biodistribution Studies

All animal studies were approved by the Ethics Committee for Animal Research in Uppsala, Sweden, following the national legislation on protection of laboratory animals (4/16, 26 February 2016).

BALB/c nu/nu female mice were implanted subcutaneously with PC-3 cells in PBS (6.5 × 10^6^ cells/mouse) on the hind leg. Tumor size was 0.4 ± 0.2 g at the time of the experiment (2−3 weeks following implantations). A group of four mice was used per data point.

One group of mice was intravenously injected with 40 pmol (30 kBq in 100 µL PBS) of [^111^In]In-DOTA-ABD-RM26 and another group was injected with the same amount and activity of [^111^In]In-DOTA-ABD-RM26 along with 10 nmol of DOTA-ABD-RM26 to test the in vivo targeting specificity for [^111^In]In-DOTA-ABD-RM26. The mice were euthanized at 72 h post injection (pi).

To study the biodistribution over time, four groups of mice were injected with 40 pmol (30 kBq in 100 µL PBS) of [^111^In]In-DOTA-ABD-RM26 and euthanized at predetermined time points of 1, 24, 72 and 144 h pi. A group of mice was injected with 40 pmol (30 kBq in 100 µL PBS) of [^111^In]In-DOTA-RM26 and euthanized at 1 h pi. After euthanization, the organs of interest were collected and weighed, and the radioactivity content was measured on a gamma counter.

## 3. Results

### 3.1. Peptide Synthesis, Purification and Conjugation

**Figure 1:**
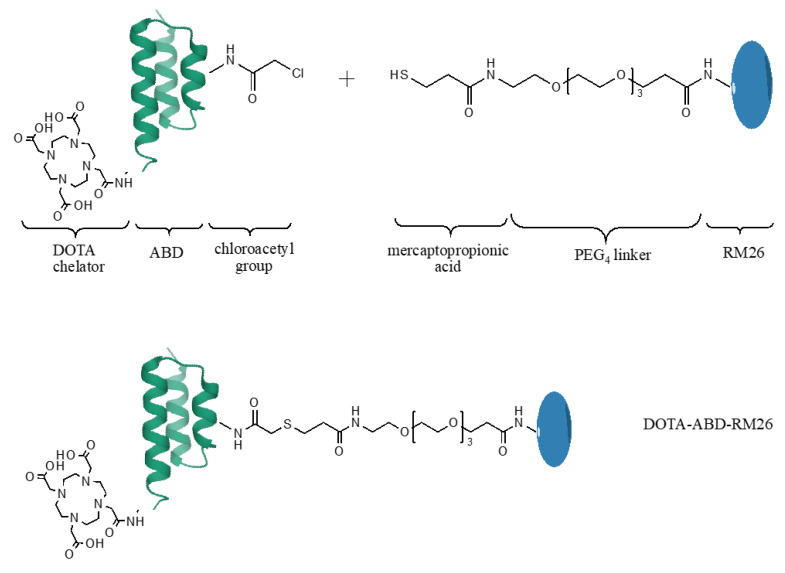
Schematic protocol for production of the DOTA-ABD-RM26 conjugate.

The syntheses of DOTA-ABD-Cl and RM26 were successfully performed in an automated SPPS system using microwave-assisted coupling and Fmoc chemistry. Double-coupling steps for the selected amino acids were introduced in order to increase the yield. Final modifications were performed manually. Following the final TFA cleavage and ether extraction of both peptides, RP-HPLC was performed to purify the crude peptides. The crude purities of DOTA-ABD-Cl and RM26 after the syntheses were 40% and 45%, respectively. The purified products were used as starting material for the conjugation reaction (Figure 1).

During the different steps of the synthesis, the peptides and the final products were analyzed, and the correct molecular weights were confirmed with MALDI-MS (Table 1; Figures S1–S3). The conjugation reaction was performed based on a previously developed protocol [26]. After the successful crosslinking of the peptides, another HPLC purification step was performed where the peaks containing the conjugate were collected and analyzed (Figure S4). The molecular weight of the final product was confirmed by ESI-MS (Table 1; Figure S5), and the high purity of the conjugate (98%) was assessed by analytical HPLC. The correct size and purity of recombinantly produced ABD035 were verified by SDS-PAGE (Figure S6) and MALDI-MS (Table 1; Figure S7).

### 3.2. Characterization of Synthesized Conjugate

**Figure 2:**
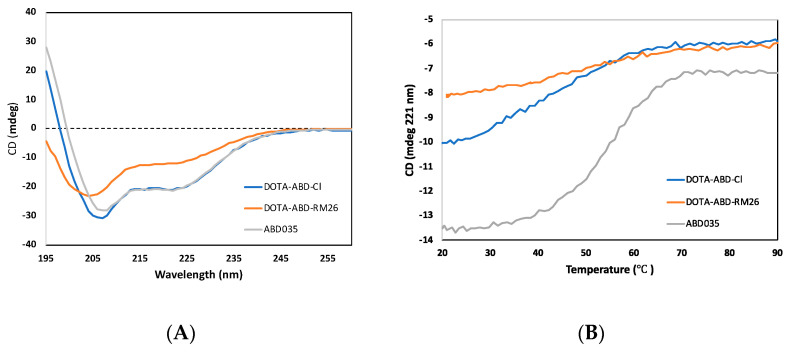
Circular dichroism (CD) spectra (**A**) and thermal melting curves (**B**) of DOTA-ABD-RM26, DOTA-ABD-Cl and ABD035.

Circular dichroism: The CD spectra of both DOTA-ABD-Cl and the control protein ABD035 displayed a characteristic alpha-helical pattern with minima at 208 nm and 222 nm (see Figure 2A). The CD spectrum of the DOTA-ABD-RM26 conjugate indicated a contribution of both random coil and alpha-helix, with a signal minimum at approximately 205 nm and a dip at 222 nm. The melting temperature was estimated to be 50 °C for the synthetic constructs DOTA-ABD-Cl and DOTA-ABD-RM26, and 54 °C for the recombinant control protein ABD035 (Figure 2B). CD spectra collected at 20 °C after thermal unfolding suggest that all proteins refold after heating.

**Figure 3:**
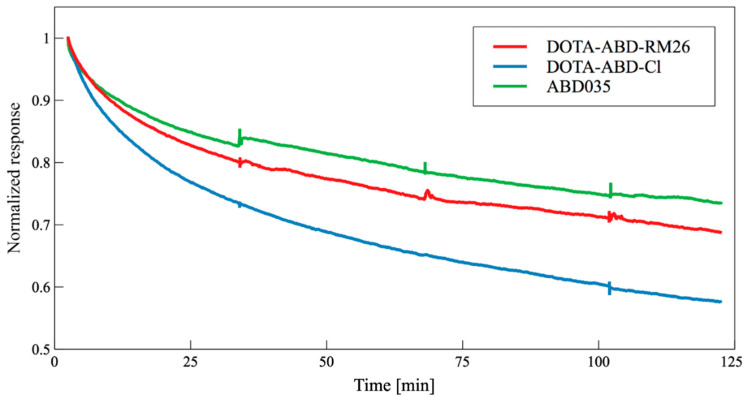
Surface plasmon resonance (SPR) sensorgrams illustrating the relative responses of DOTA-ABD-RM26, DOTA-ABD-Cl and ABD035 binding to HSA at 200 nM. Responses are normalized to the starting point of the dissociation to allow for comparison of dissociation phases for the three constructs.

SPR analyses: According to the SPR analysis of the interaction between DOTA-ABD-RM26 and HSA, the conjugation of RM26 and DOTA to ABD had a limited impact on the binding of ABD035 to HSA at room temperature (Figure 3). The K_D_ (equilibrium dissociation constant) for DOTA-ABD-RM26 binding to HSA is similar to that of the control protein ABD035 alone for binding to HSA (Table 2). SPR sensorgrams for each construct are to be found in Supplementary Information Figures S8–S10. As presented in Figure 3, the dissociation rate is the fastest for DOTA-ABD-Cl among the three constructs, while the unmodified ABD035 has the slowest dissociation rate.

### 3.3. Radiolabelling of DOTA-ABD-RM26 and Stability of the Labelled Conjugate

DOTA-ABD-RM26 was radiolabelled with indium-111 with good radiochemical yield (63 ± 3%) determined by ITLC. After purification on a size-exclusion column, the radiochemical purity of [^111^In]In-DOTA-ABD-RM26 was higher than 99.5%. The purified [^111^In]In-DOTA-ABD-RM26 was stable in PBS with minimal release of indium-111 within 24 h incubation (0.9 ± 0.2%). However, there was noticeable release of indium-111 when challenged with 1000*-fold* molar excess of EDTA—15 ± 1% release after 24 h incubation.

### 3.4. In Vitro Characterization of [^111^In]In-DOTA-ABD-RM26

**Figure 4:**
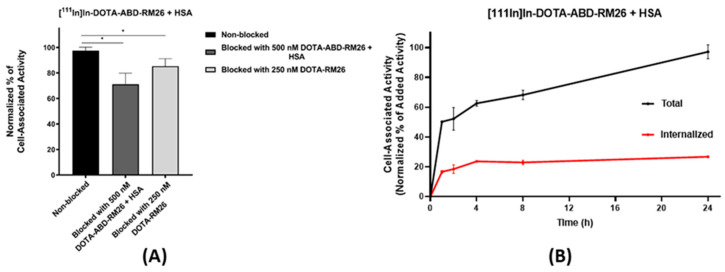
(**A**) In vitro binding specificity. (**B**) Cellular processing under continuous incubation at 1, 2, 4, 8 and 24 h. The error bars represent the standard deviation (*n* = 3). * indicates a significant difference with a *p* value < 0.05.

The binding specificity of [^111^In]In-DOTA-ABD-RM26 toward GRPR: the in vitro binding specificity assay (Figure 4A) showed significantly decreased cell-associated activity when GRPR-expressing PC-3 cells were preincubated with 250 nM of RM26 or 500 nM of DOTA-ABD-RM26 in the presence of HSA. This demonstrated that the GRPR antagonist retained specific binding toward GRPR after conjugation with ABD. This also confirmed that the conjugate bound to HSA retained binding to GRPR.

Cellular processing: The cellular processing of [^111^In]In-DOTA-ABD-RM26 was studied on GRPR-expressing PC-3 cells in the presence of 10-fold molar excess of HSA (Figure 4B). Internalization of the radiolabelled conjugate was slow, and after 24 h of incubation, no more than 27% of cell-associated activity was internalized, which corroborated the antagonistic nature of RM26.

**Figure 5:**
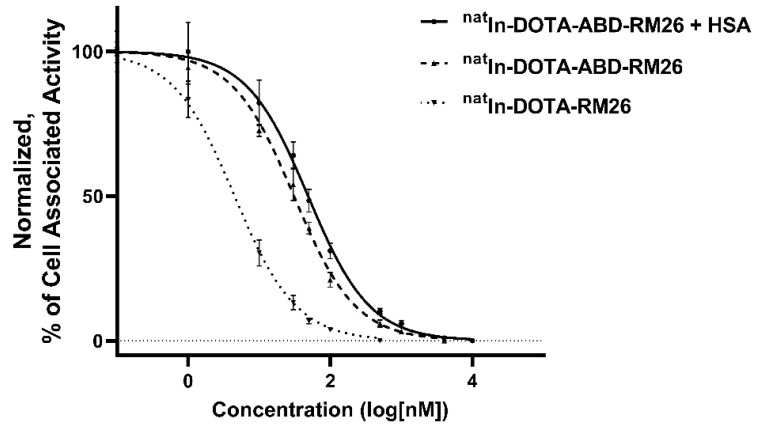
Half-maximal inhibitory concentration (IC_50_). The error bars represent the standard deviation (*n* = 3).

Competitive binding: The half-maximal inhibitory concentration (IC_50_) for ^nat^In-DOTA-ABD-RM26 was 30 ± 3 nM and 49 ± 5 nM in the presence of 10-fold molar excess of HSA, which demonstrated that binding of albumin to the ABD-containing conjugate did not influence the binding to GRPR (Figure 5). The half-maximal inhibitory concentration for ^nat^In-DOTA-RM26 measured in the same setting was 4.5 ± 0.7 nM.

### 3.5. In Vivo Characterization of [^111^In]In-DOTA-ABD-RM26

**Figure 6:**
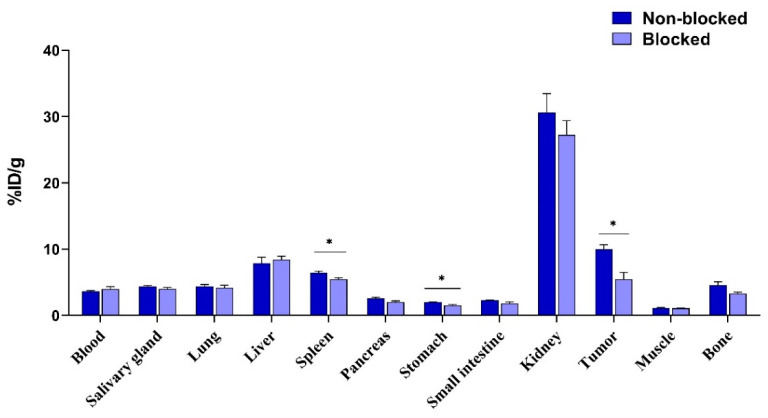
In vivo targeting specificity at 72 h pi. The error bars represent the standard deviation. * indicates a significant difference with a *p* value < 0.05.

In vivo targeting specificity: The in vivo targeting specificity of [^111^In]In-DOTA-ABD-RM26 to GRPR was studied in mice bearing PC-3 xenografts and demonstrated significant reduction of activity uptake in tumors when 10 nmol of non-labelled DOTA-ABD-RM26 was co-injected with the labelled conjugate (5 ± 2% ID/g) and compared with the group injected with 40 pmol of [^111^In]In-DOTA-ABD-RM26 (10 ± 1% ID/g) (Figure 6). The activity uptake in the spleen and stomach was slightly but significantly lower in the blocked group than in the non-blocked group.

**Figure 7:**
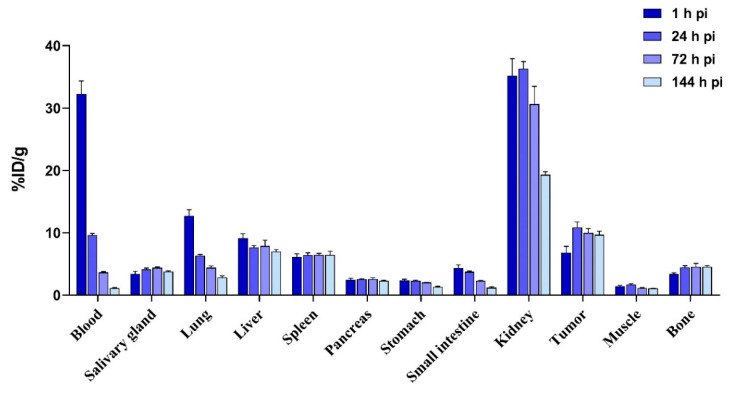
Biodistribution of [^111^In]In-DOTA-ABD-RM26 at 1, 24, 72 and 144 h pi. The error bars represent the standard deviation.

Biodistribution of [^111^In]In-DOTA-ABD-RM26: The biodistribution of [^111^In]In-DOTA-ABD-RM26 1 h pi showed the highest activity concentration in blood (32 ± 4% ID/g) and an elevated uptake in kidneys (35 ± 6% ID/g) (Figure 7). The activity uptake in PC-3 tumors was 7 ± 2% ID/g at this time point. At 24 h pi, the activity uptake in blood decreased more than 3-fold, while the activity uptake in tumors increased almost 2-fold to 11 ± 2% ID/g. At this time point, activity uptake in the lungs decreased 2-fold, but activity uptake remained stable in the majority of the other studied organs, except bones, where it increased significantly from 3.3 ± 0.4% ID/g at 1 h pi to 4.4 ± 0.5% ID/g at 24 h pi.

With time, the activity concentration in blood continued to decrease, and the activity uptake in the kidney was reduced 2-fold 6 d pi. The tumor activity uptake remained stable within the observation period (144 h pi 10 ± 1% ID/g). The activity uptake in the majority of the other organs remained stable, except the lungs and intestine where it continuously decreased.

**Figure 8:**
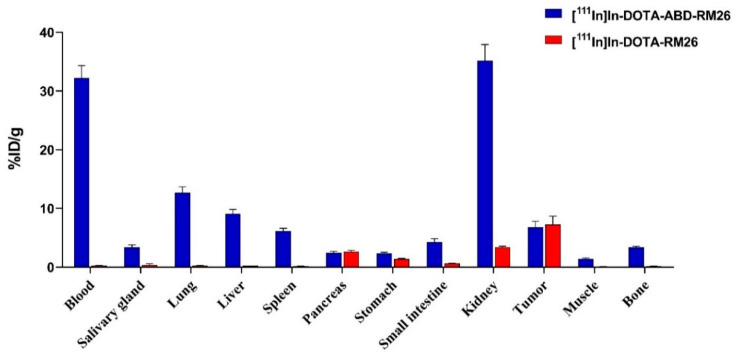
Comparison between biodistribution of [^111^In]In-DOTA-ABD-RM26 and [^111^In]In-DOTA-RM26 at 1 h pi. The error bars represent the standard deviation.

The biodistribution of [^111^In]In-DOTA-ABD-RM26 was compared with that of [^111^In]In-DOTA-RM26 1 h pi to evaluate the impact of ABD coupling on the biodistribution pattern and tumor targeting of RM26, as shown in Figure 8. The uptake in the pancreas (GRPR-expressing tissue) and tumor were on the same level for both radiolabelled conjugates. The activity uptake of [^111^In]In-DOTA-ABD-RM26 was significantly higher than that of [^111^In]In-DOTA-RM26 in all other studied organs and tissues. Activity concentration in blood was over 100-fold higher for the GRPR-targeting peptide coupled to ABD (32 ± 4% ID/g for [^111^In]In-DOTA-ABD-RM26 compared with 0.27 ± 0.04% ID/g for [^111^In]In-DOTA-RM26).

## 4. Discussion

Prostate cancer remains a challenge with high incidence and mortality rates. Several molecular targets associated with prostate cancer cells have been identified with the main focus aimed at prostate specific membrane antigen (PSMA)-targeting [27]. Targeting PSMA has demonstrated its utility in prostate cancer patient management both for diagnostic and radionuclide targeted therapy. However, other targets need to be evaluated for the treatment of prostate cancer, since not all tumors overexpress PSMA [28]. GRPR has emerged as an important target in a number of cancers including prostate cancer [20]. Targeting GRPR in oligometastatic prostate cancer could improve therapy output in early stages of prostate cancer. GRPR antagonists have been used for imaging GRPR-expressing tumors demonstrating high sensitivity and safety [8,17,18,19,29]. Recent clinical studies have commenced evaluating GRPR antagonists for treating patients with metastatic castration-resistant prostate cancer showing promising results [23].

Radiolabelled GRPR-targeting antagonistic peptides have demonstrated high and specific uptake in GRPR lesions and a rapid blood clearance which is favorable to diagnostic imaging shortly after administration, but for targeted radiotherapy, multiple injections would be required. The aim of this study was to develop a GRPR-targeting ligand based on a GRPR antagonist and an ABD that would have prolonged blood circulation time and, therefore, increase the tumor uptake of the ligand. This therapeutic construct could further be coupled to a cytotoxic agent (drug or therapeutic radionuclide) and used for targeting therapy of cancer. This approach would facilitate therapy by minimizing the number of injections and by improving delivery of cytotoxic agent to tumors.

To extend the circulatory half-life of the RM26 analogue targeting the GRPR receptor, a construct was designed with the peptide conjugated to an ABD (see Figure 1). Since the C-terminus of RM26 is important for receptor binding [30], the *N*-terminus of the peptide was modified for crosslinking to ABD. These modifications included the addition of a PEG_4_ linker as a spacer between the two molecules and a terminal thiol-containing mercaptopropionic acid residue for the conjugation to ABD via a thioether bond. ABD was functionalized with a chloroacetyl group at the Lys residue in position 14. Position 14 was chosen for conjugation as helices two and three of ABD are responsible for the interaction with HSA, and by coupling RM26 to the first helix of ABD, it was hypothesized that the HSA binding would not be impaired. In an earlier study, it has been shown that this position in ABD is suitable for conjugation of short peptides, without disrupting HSA binding [26]. The chelator DOTA was coupled to the N-terminus of ABD for the purpose of coordinating radionuclides for in vitro and in vivo experiments.

The designed conjugate, entitled DOTA-ABD-RM26, was successfully labelled with indium-111, a radiometal with relatively long half-life (2.81 d), allowing for studies of the biodistribution pattern of the labelled conjugate over several days. We noticed that both labelling yield and stability of the new conjugate were moderate when challenged with EDTA. Together this could mean sterical hindrance in building the In-DOTA cage complex, because usually this complex is very stable [31]. The new GRPR-binding conjugate retained both specific targeting to the receptor and antagonistic feature despite the bulky modification (the total molecular weight of albumin-ABD-RM26 construct is approximately 60-fold higher than for RM26). However, both coupling of ABD and further interaction of the conjugate with albumin negatively affected binding to GRPR, decreasing the IC_50_ value for ABD-RM26 towards GRPR. Further, the apparent affinity of ABD-RM26 toward HSA was slightly compromised (see Table 2). In the literature, affinity measurements of ABD035 have indicated higher affinity for HSA with a K_D_ of about 120 fM [25]. However, the very slow off-rates make it difficult to calculate the kinetic parameters, and the results presented herein should be seen as a comparison of the relative affinities of the ABD constructs rather than exact values. The obtained results confirm that the conjugation of RM26 and DOTA only marginally affects the binding of ABD035 to HSA, and that high affinity to HSA is retained, with an estimated K_D_ of 83 pM for DOTA-ABD-RM26 binding to HSA. A folded protein typically gives rise to a defined CD spectrum, while unfolding or partially unfolding the protein leads to changes in secondary structure, which results in changes in the CD spectrum. Far-UV CD spectra on DOTA-ABD-Cl and ABD035 are typical of helical proteins, while ABD-RM26 is partially unfolded (Figure 2A). According to helix–coil theory [32], ABD035 is about 75% helical (36 out of 49 residues in helical conformation), and DOTA-ABD-Cl is about 60% helical (27 out of 46 residues in helical conformation), while ABD-RM26 is only about 33% helical (18 out of 56 residues in helical conformation). A helicity of 75% for ABD035 is in agreement with a solution NMR structure of the serum albumin binding domain G148−GA3, which show a helicity of about 80% in the same region [33].

While data from our SPR measurements indicate that conjugation of DOTA and RM26 has a minimal impact on ABD binding to HSA at 25 °C, the CD spectra display lower helicity of DOTA-ABD-RM26 than ABD and DOTA-ABD-Cl under the tested conditions. When determining melting point for the three constructs, we found that T_m_ is approximately 50−54 °C for all constructs. However, as a consequence of the low amplitude for the melting curve of DOTA-ABD-RM26, the melting temperature can only be roughly estimated for this conjugate. Our data showed that DOTA-ABD-RM26 had low helicity at 42 °C in the tested conditions during CD measurements. This could have an impact on experiments performed in vivo. However, bombesin has previously been shown to form alpha-helical structures upon interaction with lipids and membranes [34]. Hence, the conditions used for measurements of T_m_ and secondary structure by CD might not be fully representative of the biophysical properties of DOTA-ABD-RM26 when interacting with GRPR or HSA in vivo.

Despite compromised affinity to GRPR, the new conjugate bound to GRPR specifically in mice bearing PC-3 xenografts. The in vivo targeting specificity was evaluated at 72 h pi so that the radioligand could be appropriately cleared from blood and other non-targeting organs. It is interesting to note that while activity uptake decreased 2-fold in xenografts with high GRPR expression, the uptake in other GRPR-expressing tissues (pancreas and organs of GI tract) decreased only marginal. Lower GRPR expression in these organs than in xenografts together with relatively moderate affinity of the new conjugate to GRPR (IC_50_ 49 ± 5 nM vs 4.5 ± 0.7 nM for parental peptide DOTA-RM26) could explain this observation.

The utilized approach for extension of blood circulation of a GRPR-targeting peptide by conjugation to ABD resulted in significantly slower blood clearance of the targeting agent. The activity concentration in blood 1 h pi was as high as 32 ± 4% ID/g and decreased only 3.5-fold at 24 h pi. Such extension would not be possible for the relatively small ABD-RM26 conjugate (7 kDa) without interaction with albumin in blood circulation [35,36,37]. High activity concentration in blood contributed to elevated activity uptake in non-targeted organs (see Figure 8). Contrastingly, in targeted organs (pancreas and intestines) and GRPR expressing xenografts, activity uptake was equal both for ABD-coupled and parental RM26. We could speculate that the initially bulky ABD-RM26/albumin complex has limited tissue penetration in comparison with the small RM26 peptide. However, the high concentration of labelled conjugate in blood overcame this limitation, and activity uptake in tumors increased up to 24 h pi, while RM26 reached maximum activity uptake at 1 h pi (with 80% of maximum uptake reaching 30 min pi, data for [^177^Lu]Lu-DOTAGA-RM26 [21]).

The activity uptake in tumors increased by 50% from 1 to 24 h pi; this demonstrated the presence of an intact radiolabelled conjugate in blood circulation capable of binding. This could be evidence that the ABD-fused GRPR antagonist escaped neprilysin activity in blood circulation. This is of special importance for targeting therapy. Furthermore, the activity uptake in tumors remained stably high up to 6 d pi, which dramatically differentiated this conjugate from small antagonistic peptides targeting GRPR. Typically, activity uptake in tumors decreases 2-4-fold within 24 h pi and continues to decrease further with time [21,22,38].

However, the in vivo study also showed a very high activity uptake in kidneys, higher than that in tumors at all studied time points. This high renal uptake was unexpected because conjugation of ABD to small proteins has been shown to decrease renal reabsorption dramatically, e.g., renal activity uptake decreased 30-fold for anti-HER2 affibody molecules when coupled to ABD and was below 5% ID/g at a peak 24 h pi [35,39]. The GRPR antagonist RM26 exhibited low renal reabsorption: for RM26 labelled with lutetium-177, the highest activity uptake in kidneys was found to be at 30 min pi and dropped 2-fold during the next 30 min [21]. Thus, neither RM26 nor ABD have a tendency to accumulate in the kidneys. The high renal reabsorption of ABD-RM26 conjugate at later time points could be explained by corrupted affinity to albumin allowing the construct to retain in blood circulation due to high albumin availability, but directing the dissociated fraction to renal clearance. The uptake in other organs such as the liver and bone was also elevated up to 144 h pi, and this can be a consequence of the radiolabel instability in vivo.

In this study, we hypothesized that conjugation of GRPR antagonist RM26 with ABD would facilitate GRPR-targeting therapy by minimizing the number of injections, increasing and prolonging the uptake of targeting agent in tumors, and keeping the healthy organs at a safe level of exposure to cytotoxic agents, especially to radiation. This study was a proof-of-principle that this approach is suitable for the development of therapeutic agents. This conjugate could be used for inhibition of GRPR-driven cell proliferation or delivering cytotoxic agents to GRPR-overexpressing tumors following previously reported concepts [20,40]. Particularly, high renal and hepatic accumulation of the anti-HER2 affibody-ABD conjugate bearing cytotoxic maytansine derivate MC-DM1 did not cause any morphological differences or signs of injury in kidneys and livers, while it demonstrated significant therapeutic effects in a preclinical therapy experiment [41]. However the unfavorable renal reabsorption abolished the possibility of using this conjugate for radionuclide therapy, which is in fact only one type of targeting therapies, due to the high absorbed dose to the kidneys, the critical organ for radio-targeting therapy. One approach is to redesign the conjugate with the aim of improving the biodistribution profile, as the position of the ABD could alter the properties of the conjugate, as previously demonstrated [42].

## 5. Conclusions

In conclusion, we herein report a successful conjugation of a GRPR antagonist and an albumin-binding domain that retained GRPR-targeting in vivo and, due to binding to albumin, resulted in a high and stable tumor uptake over several days. The developed conjugate could be used for targeted delivery of cytotoxic drugs to GRPR-expressing tumors and to keep a high therapeutic concentration of the GRPR-targeting antagonistic agent for inhibition therapy. However, this conjugate is not suitable for radionuclide therapy, and further research with the aim of improving the biodistribution profile of the ABD-RM26 conjugate is desirable.

## Figures and Tables

**Table 1:** Peptides and proteins used in the study.

Name	Peptide Sequences ^a^	Theoretical MW (Da)	Experimental MW (Da)
RM26	Mercaptopropionyl-[PEG_4_]-D-Phe-Gln-Trp-Ala-Val-Gly-His-Sta-Leu-NH_2_	1448.7	1446 ^b^
ABD035	AMALAEAKVLANRELDKYGVSDFYKRLINKAKTVEGVEALKLHILAALP	5383.4	5367 ^b^
DOTA-ABD-Cl	[DOTA]-LAEAKVLANRELDK(ClAc)YGVSDFYKRLINKAKTVEGVEALKLHILAALP-NH_2_	5571.9	5555 ^b^
DOTA-ABD-RM26	[DOTA]-LAEAKVLANRELDK(RM26)YGVSDFYKRLINKAKTVEGVEALKLHILAALP-NH_2_	6984.2	6989 ^b^ 6983.9 ^c^

^a^ RM26 amino acid sequence shown in three-letter code, and ABD amino acid sequences shown in one-letter code. PEG_4_ = 15-amino-4,7,10,13-tetraoxapentadecacanoic acid. DOTA = 1,4,7,10-tetraazacyclododecane-1,4,7,10-tetraacetic acid. ^b^ Analyzed by MALDI-MS. ^c^ Analyzed by ESI-MS.

**Table 2:** Kinetic constants for ABD variants binding to human serum albumin (HSA) determined by SPR.

Construct	k_a_ (1/Ms)	k_d_ (1/s)	K_D_ (M)
ABD035	2.5 × 10^6^	5.4 × 10^−5^	2.2 × 10^−11^
DOTA-ABD-Cl	3.0 × 10^6^	3.8 × 10^−4^	1.3 × 10^−10^
DOTA-ABD-RM26	7.9 × 10^6^	6.6 × 10^−5^	8.3 × 10^−11^

## Supplementary Materials

The following are available online at https://www.mdpi.com/1999-4923/12/10/977/s1, Figure S1: MALDI-MS spectrum of DOTA-ABD-Cl after RP-HPLC purification; Figure S2: MALDI-MS spectrum of RM26 after RP-HPLC purification; Figure S3: MALDI-MS spectrum of DOTA-ABD-RM26 after RP-HPLC purification; Figure S4: RP-HPLC elution profiles of the DOTA-ABD-RM26 conjugation reaction; Figure S5: ESI-MS spectrum of DOTA-ABD-RM26 after RP-HPLC purification; Figure S6: SDS-PAGE analysis of recombinant ABD035; Figure S7: MALDI-MS spectrum of recombinant ABD035; Figure S8: SPR sensorgram of ABD035 injected to surface with HSA at six different concentrations; Figure S9: SPR sensorgram of DOTA-ABD035-RM26 binding to HSA at seven different concentrations; Figure S10: SPR sensorgram of DOTA-ABD035-Cl binding to HSA at seven different concentrations.

## References

[1] Bray F., Ferlay J., Soerjomataram I., Siegel R.L., Torre L.A., Jemal A. (2018). Global cancer statistics 2018: GLOBOCAN estimates of incidence and mortality worldwide for 36 cancers in 185 countries. CA Cancer J. Clin..

[2] Xiao D., Wang J., Hampton L.L., Weber H.C. (2001). The human gastrin-releasing peptide receptor gene structure, its tissue expression and promoter. Gene.

[3] Markwalder R., Reubi J.C. (1999). Gastrin-releasing peptide receptors in the human prostate: Relation to neoplastic transformation. Cancer Res..

[4] Ananias H.J., van den Heuvel M.C., Helfrich W., de Jong I.J. (2009). Expression of the gastrin-releasing peptide receptor, the prostate stem cell antigen and the prostate-specific membrane antigen in lymph node and bone metastases of prostate cancer. Prostate.

[5] Körner M., Waser B., Rehmann R., Reubi J.C. (2014). Early over-expression of GRP receptors in prostatic carcinogenesis. Prostate.

[6] Beer M., Montani M., Gerhardt J., Wild P.J., Hany T.F., Hermanns T., Müntener M., Kristiansen G. (2012). Profiling gastrin-releasing peptide receptor in prostate tissues: Clinical implications and molecular correlates. Prostate.

[7] Manyak M.J. (2008). Indium-111 capromab pendetide in the management of recurrent prostate cancer. Expert Rev. Anticancer Ther..

[8] Kähkönen E., Jambor I., Kemppainen J., Lehtiö K., Grönroos T.J., Kuisma A., Luoto P., Sipilä H.J., Tolvanen T., Alanen K. (2013). In vivo imaging of prostate cancer using [68Ga]-labeled bombesin analog BAY86-7548. Clin. Cancer Res..

[9] Reynolds T.J.S., Smith C.J., Lewis M.R. (2018). Peptide-Based Radiopharmaceuticals for Molecular Imaging of Prostate Cancer. Adv. Exp. Med. Biol..

[10] Schroeder R.P.J., Müller C., Reneman S., Melis M.L., Breeman W.A.P., de Blois E., Bangma C.H., Krenning E.P., van Weerden W.M., de Jong M. (2010). A standardised study to compare prostate cancer targeting efficacy of five radiolabeled bombesin analogues. Eur. J. Nucl. Med. Mol. Imaging.

[11] Millar J.B., Rozengurt E. (1990). Chronic desensitization to bombesin by progressive down-regulation of bombesin receptors in Swiss 3T3 cells. Distinction from acute desensitization. J. Biol. Chem..

[12] Casanueva F.F., Perez F.R., Casabiell X., Camiña J.P., Cai R.Z., Schally A.V. (1996). Correlation between the effects of bombesin antagonists on cell proliferation and intracellular calcium concentration in Swiss 3T3 and HT-29 cell lines. Proc. Natl. Acad. Sci. USA.

[13] Mitran B., Tolmachev V., Orlova A. (2020). Radiolabeled GRPR Antagonists for Imaging of Disseminated Prostate Cancer. Influence of Labeling Chemistry on Targeting Properties. Curr. Med. Chem..

[14] Mitran B., Varasteh Z., Selvaraju R.K., Lindeberg G., Sörensen J., Larhed M., Tolmachev V., Rosenström U., Orlova A. (2016). Selection of optimal chelator improves the contrast of GRPR imaging using bombesin analogue RM26. Int. J. Oncol..

[15] Varasteh Z., Rosenström U., Velikyan I., Mitran B., Altai M., Honarvar H., Rosestedt M., Lindeberg G., Sörensen J., Larhed M. (2014). The effect of mini-PEG-based spacer length on binding and pharmacokinetic properties of a 68Ga-labeled NOTA-conjugated antagonistic analog of bombesin. Molecules.

[16] Varasteh Z., Mitran B., Rosenström U., Velikyan I., Rosestedt M., Lindeberg G., Sörensen J., Larhed M., Tolmachev V., Orlova A. (2015). The effect of macrocyclic chelators on the targeting properties of the 68Ga-labeled gastrin releasing peptide receptor antagonist PEG2-RM26. Nucl. Med. Biol..

[17] Zhang J., Niu G., Fan X., Lang L., Hou G., Chen L., Wu H., Zhu Z., Li F., Chen X. (2018). PET Using a GRPR Antagonist 68Ga-RM26 in Healthy Volunteers and Prostate Cancer Patients. J. Nucl. Med..

[18] Zang J., Mao F., Wang H., Zhang J., Liu Q., Peng L., Li F., Lang L., Chen X., Zhu Z. (2018). 68Ga-NOTA-RM26 PET/CT in the Evaluation of Breast Cancer: A Pilot Prospective Study. Clin. Nucl. Med..

[19] Zang J., Liu Q., Sui H., Guo H., Peng L., Li F., Lang L., Jacobson O., Zhu Z., Mao F. (2020). Combined 68Ga-NOTA-Evans Blue Lymphoscintigraphy and 68Ga-NOTA-RM26 PET/CT Evaluation of Sentinel Lymph Node Metastasis in Breast Cancer Patients. Bioconjug. Chem..

[20] Cornelio D.B., Roesler R., Schwartsmann G. (2007). Gastrin-releasing peptide receptor as a molecular target in experimental anticancer therapy. Ann. Oncol..

[21] Mitran B., Rinne S.S., Konijnenberg M.W., Maina T., Nock B.A., Altai M., Vorobyeva A., Larhed M., Tolmachev V., de Jong M. (2019). Trastuzumab cotreatment improves survival of mice with PC-3 prostate cancer xenografts treated with the GRPR antagonist 177Lu-DOTAGA-PEG2-RM26. Int. J. Cancer.

[22] Dumont R.A., Tamma M., Braun F., Borkowski S., Reubi J.C., Maecke H., Weber W.A., Mansi R. (2013). Targeted radiotherapy of prostate cancer with a gastrin-releasing peptide receptor antagonist is effective as monotherapy and in combination with rapamycin. J. Nucl. Med..

[23] Kurth J., Krause B.J., Schwarzenböck S.M., Bergner C., Hakenberg O.W., Heuschkel M. (2020). First-in-human dosimetry of gastrin-releasing peptide receptor antagonist [177Lu]Lu-RM2: A radiopharmaceutical for the treatment of metastatic castration-resistant prostate cancer. Eur. J. Nucl. Med. Mol. Imaging.

[24] Jonsson A., Dogan J., Herne N., Abrahmsén L., Nygren P.Å. (2008). Engineering of a femtomolar affinity binding protein to human serum albumin. Protein Eng. Des. Sel..

[25] Frejd F.Y., Kim K.T. (2017). Affibody molecules as engineered protein drugs. Exp. Mol. Med..

[26] Lindgren J., Refai E., Zaitsev S.V., Abrahmsén L., Berggren P.O., Karlström A.E. (2014). A GLP-1 receptor agonist conjugated to an albumin-binding domain for extended half-life. Biopolymers.

[27] Czarniecki M., Mena E., Lindenberg L., Cacko M., Harmon S., Radtke J.P., Giesel F., Turkbey B., Choyke P.L. (2018). Keeping up with the prostate-specific membrane antigens (PSMAs): An introduction to a new class of positron emission tomography (PET) imaging agents. Transl. Urol..

[28] Sheikhbahaei S., Afshar-Oromieh A., Eiber M., Solnes L.B., Javadi M.S., Ross A.E., Pienta K.J., Allaf M.E., Haberkorn U., Pomper M.G. (2017). Pearls and pitfalls in clinical interpretation of prostate-specific membrane antigen (PSMA)-targeted PET imaging. Eur. J. Nucl. Med. Mol. Imaging.

[29] Minamimoto R., Hancock S., Schneider B., Chin F.T., Jamali M., Loening A., Vasanawala S., Gambhir S.S., Iagaru A. (2016). Pilot Comparison of 68Ga-RM2 PET and 68Ga-PSMA-11 PET in Patients with Biochemically Recurrent Prostate Cancer. J. Nucl. Med..

[30] Mervic M., Moody T.W., Komoriya A. (1991). A structure function study of C-terminal extensions of bombesin. Peptides.

[31] Wadas T.J., Wong E.H., Weisman G.R., Anderson C.J. (2010). Coordinating Radiometals of Copper, Gallium, Indium, Yttrium, And Zirconium for Pet and Spect Imaging of Disease. Chem. Rev..

[32] Scholtz J.M., Qian H., York E.J., Stewart J.M., Baldwin R.L. (1991). Parameters of helix-coil transition theory for alanine-based peptides of varying chain lengths in water. Biopolymers.

[33] Johansson M.U., Frick I.M., Nilsson H., Kraulis P.J., Hober S., Jonasson P., Linhult M., Nygren P.Å., Uhlén M., Björck L. (2002). Structure, specificity, and mode of interaction for bacterial albumin-binding modules. J. Biol. Chem..

[34] Cavatorta P., Farruggia G., Masotti L., Sartor G., Szabo A.G. (1986). Conformational flexibility of the hormonal peptide bombesin and its interaction with lipids. Biochem. Biophys. Res. Commun..

[35] Tolmachev V., Orlova A., Pehrson R., Galli J., Baastrup B., Andersson K., Sandström M., Rosik D., Carlsson J., Lundqvist H. (2007). Radionuclide therapy of HER2-positive microxenografts using a 177Lu-labeled HER2-specific Affibody molecule. Cancer Res..

[36] Kelly J.M., Amor-Coarasa A., Ponnala S., Nikolopoulou A., Williams C., DiMagno S.G., Babich J.W. (2019). Albumin-Binding PSMA Ligands: Implications for Expanding the Therapeutic Window. J. Nucl. Med..

[37] Garousi J., von Witting E., Borin J., Vorobyeva A., Altai M., Vorontsova O., Konijnenberg M.W., Oroujeni M., Orlova A., Tolmachev V. (2020). Radionuclide Therapy Using ABD-fused ADAPT Scaffold Protein: Proof of Principle. Biomaterials.

[38] Dalm S.U., Bakker I.L., de Blois E., Doeswijk G.N., Konijnenberg M.W., Orlandi F., Barbato D., Tedesco M., Maina T., Nock B.A. (2017). 68Ga/177Lu-NeoBOMB1, a Novel Radiolabeled GRPR Antagonist for Theranostic Use in Oncology. J. Nucl. Med..

[39] Orlova A., Jonsson A., Rosik D., Lundqvist H., Lindborg M., Abrahmsen L., Ekblad C., Frejd F.Y., Tolmachev V. (2013). Site-specific radiometal labeling and improved biodistribution using ABY-027, a novel HER2-targeting affibody molecule-albumin-binding domain fusion protein. J. Nucl. Med..

[40] Safavy A., Raisch K.P., Khazaeli M.B., Buchsbaum D.J., Bonner J.A. (1999). Paclitaxel Derivatives for Targeted Therapy of Cancer: Toward the Development of Smart Taxanes. J. Med. Chem..

[41] Altai M., Liu H., Ding H., Mitran B., Edqvist P.H., Tolmachev V., Orlova A., Gräslund T. (2018). Affibody-derived drug conjugates: Potent cytotoxic molecules for treatment of HER2 over-expressing tumors. J. Control. Release.

[42] Altai M., Leitao C.D., Rinne S.S., Vorobyeva A., Atterby C., Ståhl S., Tolmachev V., Löfblom J., Orlova A. (2018). Influence of Molecular Design on the Targeting Properties of ABD-Fused Mono- and Bi-Valent Anti-HER3 Affibody Therapeutic Constructs. Cells.

