# Supplementary material for: Preclinical Evaluation of the GRPR-Targeting Antagonist RM26 Conjugated to the Albumin-Binding Domain for GRPR-Targeting Therapy of Cancer
Source: Pharmaceutics. 2020 Oct 16;12(10):977. doi: 10.3390/pharmaceutics12100977 (PMC7594083; doi:10.3390/pharmaceutics12100977)
Supplement: Supplementary file 1 [file pharmaceutics-12-00977-s001.zip › pharmaceutics-965072-supplementary.docx]

Supplementary Materials: Preclinical Evaluation of the GRPR-Targeting Antagonist RM26 Conjugated to Albumin-Binding Domain for GRPR-Targeting Therapy of Cancer

Ayman Abouzayed, Hanna Tano, Ábel Nagy, Sara S. Rinne, Fadya Wadeea, Sharmishtaa Kumar, Kristina Westerlund, Vladimir Tolmachev, Amelie Eriksson Karlström and Anna Orlova

**Figure S1*.*** MALDI-MS spectrum of DOTA-ABD-Cl after RP-HPLC purification (theoretical MW: 5571.9 Da, experimental MW: 5555 Da).

**Figure S2.** MALDI-MS spectrum of RM26 after RP-HPLC purification (theoretical MW: 1448.7 Da; experimental MW: 1446 Da).

**Figure S3.** MALDI-MS spectrum of DOTA-ABD-RM26 after RP-HPLC purification (theoretical MW: 6984.2 Da; experimental MW: 6989 Da).

**Figure S4.** RP-HPLC elution profiles of the DOTA-ABD-RM26 conjugation reaction (blue curve: signal 220 nm; red curve: signal 280 nm; green curve: Solvent Ratio B; pink curve: baseline).


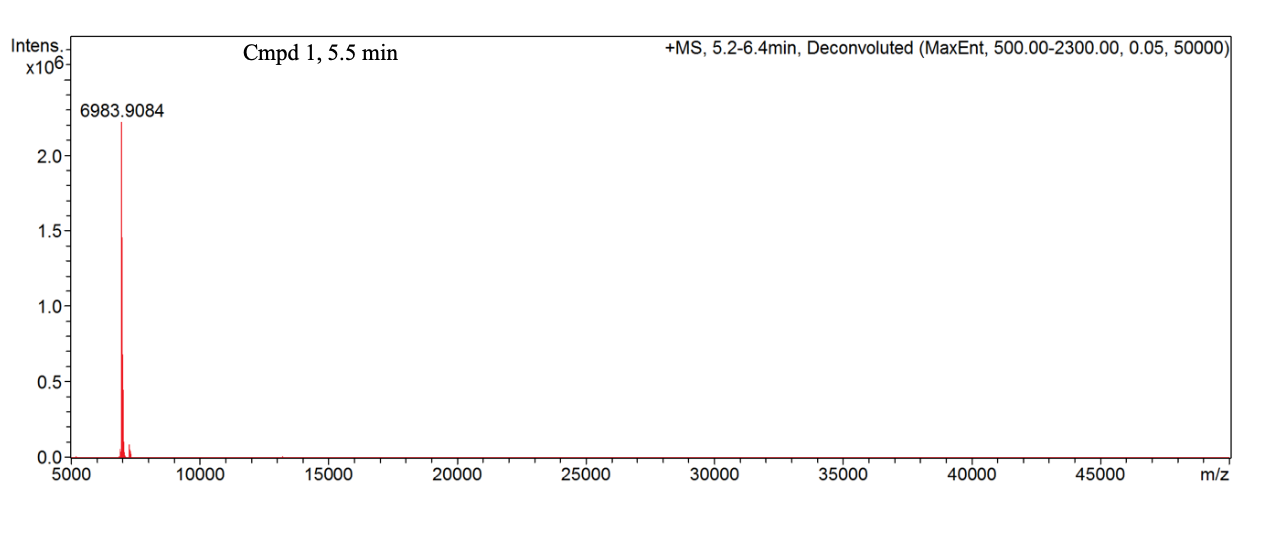


**Figure S5.** ESI-MS spectrum of DOTA-ABD-RM26 after RP-HPLC purification (theoretical MW: 6984.2 Da; experimental MW: 6983.9 Da).

1. Expression and Purification of Recombinant ABD Control Protein

ABD035 was cloned into vector pET32a(+) by introducing restriction site NcoI and XhoI. The final plasmid Trx-His6-ABD035_pET32a was transformed into *E. coli* BL21 chemically competent cells (Life Technologies), and subsequently cultivated in autoinduction media ZYP-20052S and carbenicillin at a concentration of 100 ug/ml (Studier, 2005). Following inoculation, the Trx-His6-ABD035_pET32a culture was kept at 37 °C for 4–5 h at 150 rpm and was then moved to 25 °C for 16 h at 150 rpm. Cells were harvested by centrifugation for 10 min at 4000 g and were subsequently dissolved in IMAC binding buffer (50 mM phosphate buffer, 300 mM NaCl, 10 mM imidazole pH 7.4) and lysed by sonication. Lysate was centrifuged at 16,000 g for 20 min and supernatant was subsequently subjected to IMAC purification on matrix (HisPur Cobalt Resin, Thermo Scientific). Trx-His6-ABD035 was purified using binding/wash buffer (50 mM phosphate buffer, 300 mM NaCl, 10 mM imidazole pH 7.4) and elution buffer (50 mM phosphate buffer, 300 mM NaCl, 300 mM imidazole pH 7.4). Eluted Trx-His6-ABD035 was buffer exchanged to 10 mM NaAc pH 4.6 using a PD-10 desalting column (GE Healthcare) and was subsequently lyophilized.

Trx-His6-enterokinase-ABD035 was cleaved by enterokinase-His_6_ (Sino Biological) dissolved in 50 mM Tris-HCl pH 7.5. 9.2 mg Trx-His6-enterokinase-ABD035 at a volume of 3.5 mL was incubated with 15 ul enterokinase at a concentration of 0.15 mg/ml for 2 h at 37 °C. Following cleavage, the enterokinase and cleaved Trx-His6-enterokinase-ABD035 were subjected to pre-equilibrated IMAC matrix and ABD035 were collected from the flow through after incubation for 30 min shaking at room temperature. Buffer was changed from 50 mM Tris-HCl pH 7.5 to 10 mM NH_4_Ac using Amicon Ultra-15 spin column 300 Da cut-off (Merck) and ABD035 were subsequently lyophilized. Purity and molecular weight of purified and cleaved protein was confirmed using MALDI-TOF and SDS-PAGE.


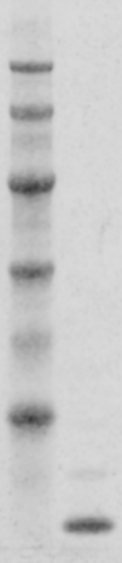


**Figure S6.** SDS-PAGE analysis of recombinant ABD035. Left lane consists of ladder (Amersham Low Molecular Weight Calibration for SDS Electrophoresis, GE Healthcare) with molecular weights marked at 14.4, 20.1, 30, 45, 66 and 97 kDa.

**Figure S7.** MALDI-MS spectrum of recombinant ABD035 (theoretical MW: 5384.2 Da; experimental MW: 5367 Da).


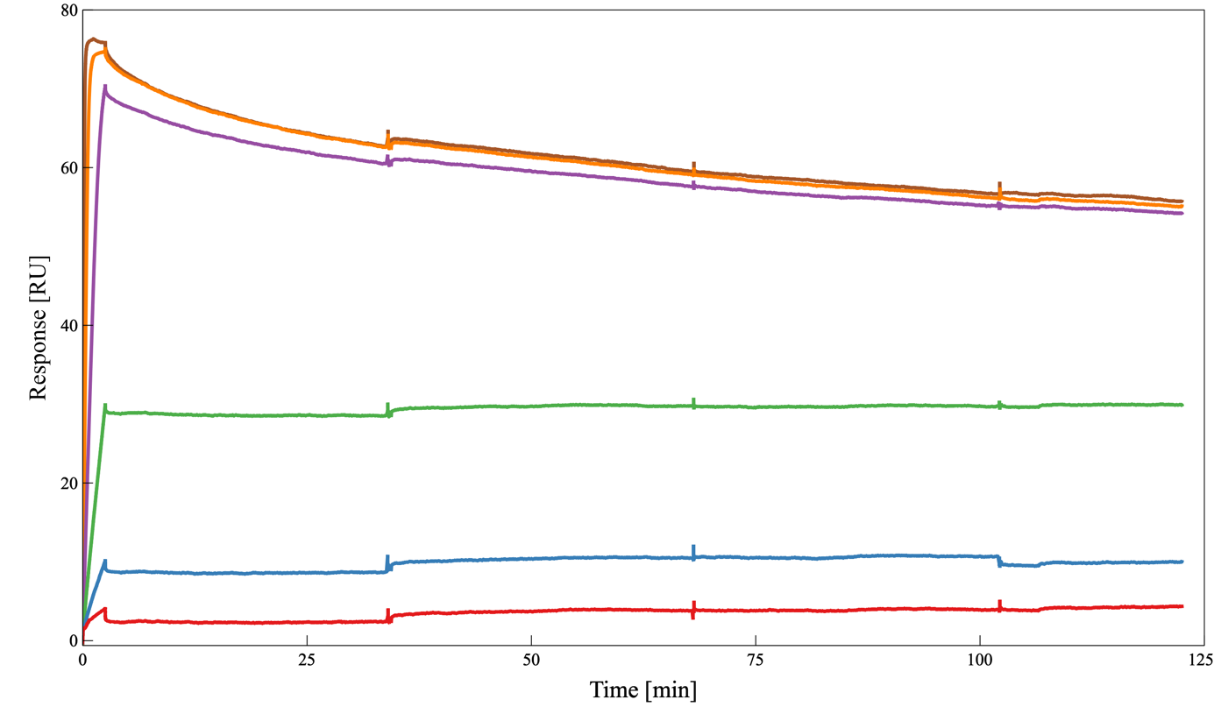


**Figure S8.** SPR sensorgram of ABD035 injected to surface with HSA at 6 different concentrations (0.82, 2.47, 7.4, 22.2, 66.7 and 200 nM).


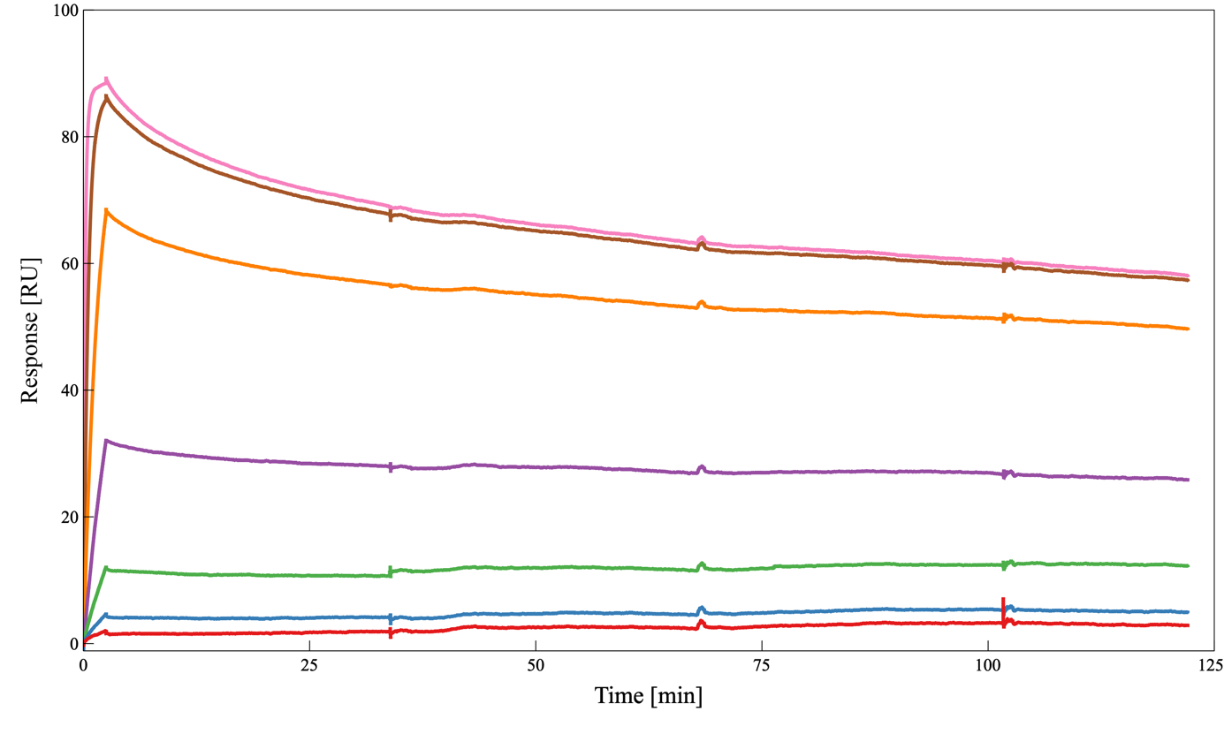


**Figure S9.** SPR sensorgram of DOTA-ABD035-RM26 binding to HSA at 7 different concentrations (0.27, 0.82, 2.47, 7.4, 22.2, 66.7 and 200 nM).


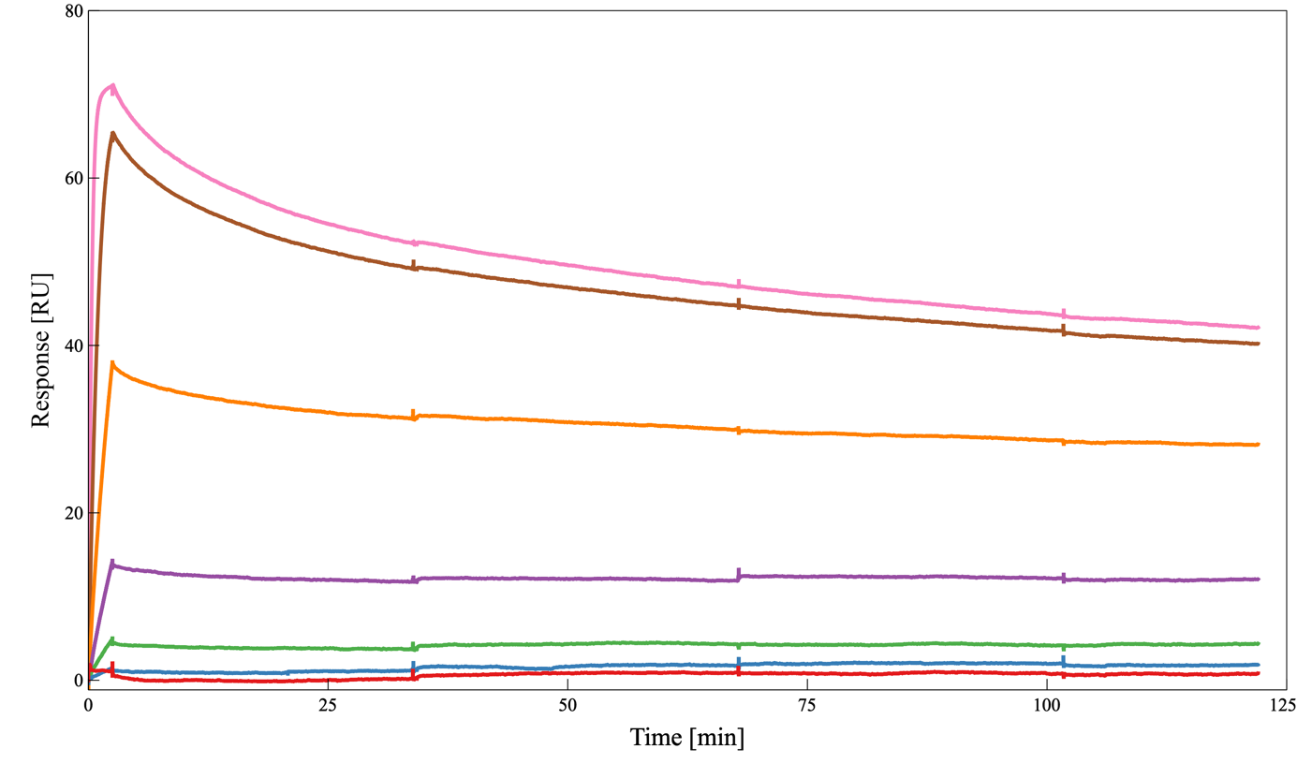


**Figure S10*.*** SPR sensorgram of DOTA-ABD035-Cl binding to HSA at 7 different concentrations (0.27, 0.82, 2.47, 7.4, 22.2, 66.7 and 200 nM).
